# A regulatory circuit comprising GATA1/2 switch and microRNA-27a/24 promotes erythropoiesis

**DOI:** 10.1093/nar/gkt848

**Published:** 2013-09-18

**Authors:** Fang Wang, Yong Zhu, Lihua Guo, Lei Dong, Huiwen Liu, Haixin Yin, Zhongzu Zhang, Yuxia Li, Changzheng Liu, Yanni Ma, Wei Song, Aibin He, Qiang Wang, Linfang Wang, Junwu Zhang, Jianxiong Li, Jia Yu

**Affiliations:** ^1^Department of Biochemistry, Institute of Basic Medical Sciences, Chinese Academy of Medical Sciences (CAMS) & Peking Union Medical College (PUMC), Beijing 100005, PR China, ^2^Zebrafish Core Facility, State Key Laboratory of Medical Molecular Sciences, Institute of Basic Medical Sciences, CAMS & PUMC, Beijing 100005, PR China, ^3^Department of Biomedical Engineering, School of Computation and Information Technology, Beijing Jiaotong University, Beijing 100044, PR China, ^4^Department of Cardiology, Children’s Hospital Boston, Boston, MA 02115, USA, ^5^Institute of Zoology, Chinese Academy of Sciences, Beijing 100101, China and ^6^Cancer Center, The General Hospital of the People’s Liberation Army, Beijing 100853, PR China

## Abstract

Transcriptional networks orchestrate complex developmental processes, and such networks are commonly instigated by master regulators for development. By now, considerable progress has been made in elucidating GATA factor-dependent genetic networks that control red blood cell development. Here we reported that GATA-1 and GATA-2 co-regulated the expression of two microRNA genes, microRNA-27a and microRNA-24, with critical roles in regulating erythroid differentiation. In general, GATA-2 occupied the miR-27a∼24 promoter and repressed their transcription in immature erythroid progenitor cells. As erythropoiesis proceeded, GATA-1 directly activated miR-27a∼24 transcription, and this involved a GATA-1-mediated displacement of GATA-2 from chromatin, a process termed ‘GATA switch’. Furthermore, the mature miR-27a and miR-24 cooperatively inhibited GATA-2 translation and favoured the occupancy switch from GATA-2 to GATA-1, thus completing a positive feedback loop to promote erythroid maturation. In line with the essential role of GATA factors, ectopic expression of miR-27a or miR-24 promoted erythropoiesis in human primary CD34+ haematopoietic progenitor cells and mice, whereas attenuated miR-27 or miR-24 level led to impaired erythroid phenotypes in haematopoietic progenitor cells and zebrafish. Taken together, these data integrated micro RNA expression and function into GATA factor coordinated networks and provided mechanistic insight into a regulatory circuit that comprised GATA1/2 switch and miR-27a/24 in erythropoiesis.

## INTRODUCTION

Erythropoiesis is the process by which haematopoietic stem/progenitor cells give rise to lineage-committed erythroid precursors, which then terminally differentiate into mature circulating red blood cells. Foundational studies have shown that the developmental regulator GATA family (especially GATA-1 and GATA-2) are involved in erythropoiesis. GATA-1 and GATA-2 are often expressed in overlapping and reciprocal patterns during erythropoiesis. The unique expression patterns of GATA-1 and GATA-2 reflect distinct biological functions; GATA-1 is essential for erythropoiesis, whereas GATA-2 regulates the development and function of haematopoietic progenitor cells (HPCs). Evidence supports a model in which these two GATA factors cross-regulate transcription of their respective genes through a process termed a GATA switch. GATA-2 is expressed earlier than GATA-1 during erythropoiesis and occupies multiple GATA sites (1–3). As GATA-1 is activated, it competes with and displaces GATA-2 occupancy from chromatin sites (4). This switch generally causes a change in transcriptional output that leads to terminal erythroid differentiation. Furthermore, it has also been proposed that this switch is essential for the expression of a large subset of erythroid genes in both human and mice.

So far, a number of non-coding regulators such as miR-451 (5,6), miR-23a (7), miR-221/222 (8), miR-376a (9) and miR-223 (10) were reported to play positive or negative roles in controlling erythropoiesis. Despite the fact that miR-451, miR-23a and mir-223 were shown to suffer from GATA-1 regulation in some species (6,7,11), there are virtually no data about GATA-1/2 switch dynamically operating on their genes during erythropoiesis. Recently, we reported that miR-23a was a positive erythroid regulator and activated by GATA-1 along erythroid differentiation (7). As miR-23a, miR-27a and miR-24-2 derive from a common gene cluster, the functional and regulatory significance of miR-27a and miR-24 reasonably needs to be further investigated. Here, we demonstrate that the GATA-1/2 switch occurs at the common gene locus encoding miR-23a, miR-27a and miR-24. We further find that both micro RNAs (miRNAs) directly co-target GATA-2 and facilitate the switch from GATA-2 to GATA-1 in the same chromatin occupancy during erythroid differentiation. Therefore, miR-27a/24 and GATA-1/2 form a regulatory circuit that supports the activation of their own genes. Meanwhile, *in vitro* and *in vivo* functional analyses indicated that miR-27a and miR-24 promoted erythroid differentiation in CD34+ HPCs, zebrafish and mice. Our study demonstrates that GATA factors elaborately control the transcription of *miR-27a* and *miR-24* and reveals a regulatory circuit that regulates the GATA-1/2 switch via miR-27a and miR-24 to promote erythroid maturation.

## MATERIALS AND METHODS

### Cell isolation and culture

Human erythroleukemia cell line K562 was maintained in RPMI1640 supplemented with 10% fetal bovine serum (Gibco, Carlsbad, CA, USA). Erythroid differentiation of K562s was obtained using 30 µM hemin (Sigma-Aldrich, Deisenhofen, Germany) over 0, 24, 48 and 72 h. The 293T cells were obtained from American Type Culture Collection and grown in DMEM media with 10% FBS. Human umbilical cord blood was obtained from normal full-term deliveries after informed consent as approved by the Research Ethics Committee of Peking Union Hospital (Beijing, China). Mononuclear cell fractions were isolated from umbilical cord blood by Percoll density gradient (d = 1.077; Amersham Biotech, Germany). CD34+ cells were enriched from mononuclear cells through positive immunomagnetic selection (CD34 MultiSort kit, Miltenyi Biotec, Bergisch-Glad-bach, Germany). The isolated CD34+ cells were cultured in IMDM supplemented with 30% fetal bovine serum (Hyclone), 1% BSA, 100 µM 2-ME, 2 ng/ml recombinant human IL-3, 100 ng/ml recombinant human SCF (Stem Cell Technologies, Vancouver, BC, Canada), 2 U/ml recombinant human EPO (R&D Systems, Minneapolis, MN, USA), 60 mg/ml penicillin and 100 mg/ml streptomycin. Cells were harvested every 3–5 days. The colony-forming cell assay was performed in triplicate using human methylcellulose media (R&D Systems, MN, USA) as described (8). For morphological analysis, cells were smeared on glass slides by centrifugation, stained with May-grünwald/Giemsa and analysed at 400× magnification under a microscope (Nikon TE2000) equipped with a digital camera.

### Oligonucleotides, plasmid and virus production

miRNA mimics (miR-27 a and miR-24), miRNA inhibitors (Anti-27a and Anti-24) and negative control molecules (Scramble) were obtained from Dharmacon (Austin, TX, USA) and transfected with DharmFECT1 (Dharmacon, Austin, TX, USA) at a final concentration of 60 nM. siRNAs smart pools (specifically for GATA-1 and GATA-2) and control siRNAs were synthesized by Dharmacon and transfected (100 nM) using DharmFECT1. The reverse complementary sequence to miR-27a or miR-24 was inserted into pGL3 downstream of the firefly luciferase gene (Promega, WI, USA) to generate a reporter construct (miR-27a Reporter or miR-24 Reporter) as a positive control in luciferase assay. The 3′ UTR of human GATA-2 mRNA was PCR amplified and cloned into pGL3 downstream of the firefly luciferase gene to generate the GATA-2 reporter. Mutations in GATA-2 mRNA sequence were created using the QuickChangeSiteDirected Mutagenesis kit (Stratagene, CA, USA). For GATA-1 or GATA-2 overexpression, full-length cDNA of GATA-1 or GATA-2 was cloned into pcDNA3.1 vectors. The primers were listed in Supplementary Table S1. For functional analysis of miRNA promoter, the wild-type and mutant constructs containing promoter region from human genomic DNA were cloned into pGL-3 basic vector upstream of the firefly gene. The self-inactivating transfer vector plasmid containing miR-27a or 24 (pMIR-27a or 24) or antisense RNAs to miR-27a or 24 (pZip-27a or 24) and the packaging kit were purchased from System Biosciences (SBI, CA, USA) and operated according to the manufacturer’s instructions. The shRNA lentivirus plasmid carrying siRNAs specific to GATA-1 was purchased from Santa Cruz Biotechnology (Santa Cruz, CA, USA) and operated according to the manufacturer’s instructions. The harvested viral particles (Lenti-27a, 24; Zip-27a, 24; or Lenti-si_GATA-1) were added to the CD34+ HPCs. Cells were washed the next day with PBS and plated for colony-forming experiments and liquid cultures.

### RNA isolation and quantitative real-time PCR

Total RNA was extracted from the cell harvest using Trizol reagent (Invitrogen, Carlsbad, CA, USA) according to manufacturer’s instruction. The RNA was quantified by absorbance at 260 nm. cDNA was synthesized by M-MLV reverse transcriptase (Invitrogen) from 2 µg of total RNA. Oligo (dT) 18 were used as the RT primers for reverse transcription of mRNAs. Quantitative real-time PCR was carried out in BIORAD IQ5 real-time PCR System (Biorad, Foster City, CA, USA) using SYBR Premix Ex Taq kit (Takara, Dalian, China) according to manufacturer’s instruction. For mRNAs, the data were normalized using the endogenous GAPDH control. For measurement of Pri-miR-27a∼24-2, miR-27a and 24 expression, q-PCR was performed using Taqman probes (Applied Biosystems, Foster City, CA, USA): pri-miR-27a∼24 (Hs03294931_pri), miR-27a (TM408), miR-24 (TM402), human GAPDH (Hs9999905_M1), RNU6B (TM1093) according to manufacturer’s instruction. Quantitative chromatin immunoprecipitation (ChIP) analysis was performed starting from 1 µl of template DNA in 20 µl of reactions. The data were presented as fold change or enrichment of precipitated DNA associated with the GATA-1 or GATA-2 relative to input chromatin. The comparative Ct method was used to quantify target genes relative to endogenous control. For each individual analysis, one of the samples was designated as the calibrator and given a relative value of 1.0. All quantities were expressed as n-fold relative to the calibrator. The primers used for PCR are listed in Supplementary Table S1.

### Northern and western blot analysis

Northern blot analysis of miRNAs was done as described (9). The oligonucleotide probe sequences are listed in Supplementary Table S1. Whole-cell lysate or nuclear extract was subjected to western blot analysis as detailed elsewhere (9). The following antibodies were used for western blot. GAPDH were purchased from Santa Cruz Biotechnology. GATA-1 (ab11963) and GATA-2 (ab22849) was purchased from Abcam Company. Immunoblots were quantified by ImageJ software.

### Luciferase reporter assay

For miRNA targets analysis, the 293T cells were co-transfected with 0.4 µg of the reporter construct, 0.02 µg of pRL-TK control vector and 5 pmol of miRNA mimic or scramble controls. For functional analysis of miRNA cluster promoter, the 293T cells were co-transfected with 0.5 µg of pGL-3 constructs containing miRNA promoter, along with 0.02 µg of pRL-TK control vector and 0.2 µg of pcDNA3.1 constructs with full-length cDNA of GATA-1, GATA-2 or empty pcDNA3.1 vector. Cells were harvested 48 h post-transfection and assayed with Dual Luciferase Assay (Promega, WI, USA) according to manufacturer’s instructions. All transfection assays were carried out in triplicate.

### ChIP assay

Antibodies Anti-GATA-1 (ab11963,) Anti-GATA-2 (ab22849) and Pol II (ab5408), purchased from Abcam Company, were used for ChIP studies. K562s induced by hemin for indicated time point were collected and cross-linked with 1% formaldehyde for 10 min, washed in cold PBS buffer, resuspended in lysis buffer [0.1% SDS, 0.5% Triton X-100, 20 mM Tris–HCl (pH 8.1), 150 mM NaCl, protease inhibitor, Roche] and sonicated to obtain chromatin fragments between 200 and 1000 bp. Sonicated chromatin was resuspended in IP buffer and incubated overnight at 4°C with magnetic beads conjugated antibodies (Santa Cruz Biotechnologies). The IP was then washed with lysis buffer, LiCl buffer [0.25 M LiCl, 1% NP-40, 1% deoxycholate, 1 mM EDTA, 10 mM Tris–HCl (pH 8.1)] and TE buffer, eluted in elution buffer (1% SDS, 0.1 M NaHCO3). The DNA was then recovered by reversing the cross-links and purified by QIAGEN Purification Kit. An un-enriched sample of DNA was treated in a similar manner to serve as input.

### Flow cytometry

Cells were harvested at indicated times and washed twice at 4°C in PBS/0.5% BSA to block Fc receptors. Transduced CD34+ HPCs were assessed for green fluorescence (GFP) and CD235a expression after staining with PE-conjugated anti-CD235a antibodys (BD Biosciences PharMingen). Mice cells were stained with PE-conjugated anti-Ter119 and FITC-conjugated anti-CD71 antibodies (eBioscience, CA, USA). Flow cytometry was carried out on a C6 Flow Cytometer® Instrument (BD Biosciences, Franklin Lakes, NJ, USA).

### *In vivo* functional analysis of miRNAs in zebrafish

Zebrafish were raised and maintained by previously described standard methods (12). Total RNA was isolated from the fertilized eggs at different stages using Trizol reagent (Invitrogen, Carlsbad, CA, USA) according to manufacturer’s instruction. Morpholinos (Gene Tools, LLC, Philomath, OR, USA) were injected into the yolk of one-cell stage embryos at a dose of 8 ng. Whole-mount *in situ* hybridizations were carried out as previously described by use of digoxigenin-labelled riboprobes for hbbe3, scl and gata-1 at indicated times (13). Haemoglobin staining with o-dianisidine was performed on morpholino (MO)-injected embryos at 48 hpf, and the embryos were then dechorionated and fixed with 4% paraformaldehyde overnight. Fixed embryos were washed in PBST for three times and then incubated in the staining buffer [0.6 mg/ml o-dianisidine, 10 mM sodium acetate (pH 5.2), 0.65% hydrogen peroxide and 40% ethanol] for 15 min in the dark. For morphological analysis, stained embryos were analysed at 400× magnification under a microscope (Nikon TE2000) equipped with a digital camera.

### Mice and transplantation assays

All animal experiments were performed with the approval of the Research Ethics Committee of Peking Union Hospital. Bone marrow cells were obtained from 6- to 8-week-old male C57Bl/6 mice that were injected intravenously with 5 mg of 5-fluorouracil. Briefly, bone marrow was flushed from femurs and tibias, and red blood cells were lysed (RBCL buffer, Sigma, CA, USA). Cells were cultured overnight with 2 ng/ml IL-3, 10 ng/ml IL-6 and 100 ng/ml SCF (Stem Cell Technologies, Vancouver, BC, Canada) in IMDM supplemented with 30% FBS. After two rounds of spin-infection with lentiviral supernatant (Genechem, Shanghai, China), these cells were washed and resuspended in PBS and were then injected into the lateral tail vein of lethally irradiated (2 × 450 cGy) male NOD/SCID recipient mice. Our results are from three mice per group. Mice were killed after 8 weeks post-transplantation. Blood, bone marrow and spleen were harvested and processed into single-cell suspensions. Samples of spleen were fixed in 10% neutral buffered formalin for paraffin sectioning.

### Statistics

Student’s *t*-test (two-tailed) was performed to analyse the data. *P* < 0.05 were considered significantly as indicated by asterisk (*P < 0.05; **P < 0.01).

## RESULTS

### Primary and mature transcripts of the *miR-23a∼27a∼24-2* cluster were upregulated in differentiated erythroid cells

**Figure 1.**
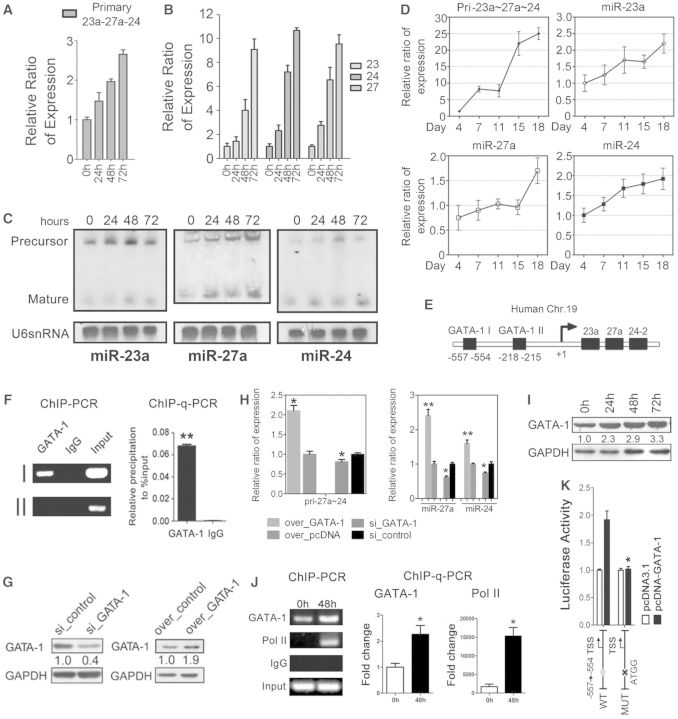
GATA-1 was located on the upstream of *miR-23a∼27a∼24-2 cluster* and activated its expression during erythropoiesis. (**A**) Q-PCR analysis of the primary transcript of the *miR-23a∼27a∼24-2 cluster* in K562s undergoing erythroid differentiation caused by hemin treatment for 0, 24, 48 and 72 h. (**B**) Q-PCR analysis of the mature transcripts of miR-27a, miR-24 and miR-23a in K562s undergoing erythroid differentiation caused by hemin treatment for 0, 24, 48 and 72 h. (**C**) Northern blot analysis of miR-27a, miR-24 and miR-23a in K562s undergoing erythroid differentiation. U6 snRNA was used as a loading control. (**D**) Q-PCR analysis of mature miR-27a and miR-24 expression in CD34+ HPCs undergoing day 4, 7, 11, 15 and 18 of E culture. (**E**) Representation of the human −603 bp *miR-23a∼27a∼24 cluster* promoter fragments. (**F**) ChIP-PCR and ChIP-qPCR analysis of the GATA-1 hit on the −557 site at the *miR-23a∼27a∼24 cluster* promoter in K562s. ChIP-q-PCR results are shown as fold enrichment compared with input. Error bars represent the standard deviation obtained from three independent experiments. **P < 0.01. (**G**) Immunoblot analysis of GATA-1 expression in K562s treated with siRNAs specific to GATA-1 (si_GATA-1) for 48 h or K562s transfected with a construct overexpressing GATA-1 (over_GATA-1) for 48 h, respectively. (**H**) Q-PCR analysis of Pri-27a∼24, mature miR-27a and miR-24 expression in K562s treated as described in (G). (**I**) Immunoblot analysis of GATA-1 expression in K562s undergoing erythroid differentiation. (**J**) ChIP-PCR and ChIP-q-PCR analysis of GATA-1 and pol II occupancy at the −557 site in hemin-treated K562s at 0 and 48 h. ChIP-q-PCR results are shown as fold enrichment compared with input. Error bars represent the standard deviation obtained from three independent experiments. *P < 0.05. (**K**) Functional activity of GATA-1 on the wild-type GATA site (WT) or mutant site (MUT) of the *miR-23a∼27a∼24* promoter in a luciferase reporter analysis. Error bars represent the standard deviation obtained from three independent experiments. *P < 0.05.

The *miR-23a∼27a∼24-2 cluster* encodes a single primary transcript composed of 3 miRNAs: miR-23a, miR-27a and miR-24. Accumulated evidence has indicated that clustered genes derived from a common ancestor often tend to possess similar features in cellular processes. Given the significant associations of miR-23a in erythropoiesis demonstrated in our previous studies (7), we decided to examine the primary and mature products from *miR-23a∼27a∼24-2 cluster.* Q-PCR using Taqman probes was performed to measure the level of primary transcripts of the *miR-23a∼27a∼24-2 cluster* and indicated that *pri-miR-23a∼27a∼24-2* was continuously increased in differentiated K562s treated with hemin (Figure 1A). Additionally, mature miR-27a and 24 were also increased in hemin-treated K562s (Figure 1B). To further validate our findings, a northern blot was performed and showed that both precursor and mature levels of miR-27a, 24 and 23a were upregulated during erythroid differentiation (Figure 1C). Furthermore, q-PCR using specific Taqman probes revealed that *pri-miR-23a∼27a∼24-2* and mature miR-27a, miR-24 and miR-23a were increased in EPO-driven erythroid differentiation of primary cultured human CD34+ HPCs (Figure 1D). Collectively, the expression patterns of miR-27a and miR-24 in two separate erythroid differentiation models (K562s and HPCs) suggested that they may be two potential regulators of erythroid differentiation.

### GATA-1 was located on the *miR-23a∼27a∼24-2* cluster promoter and activated its transcription

In our attempt to investigate the potential factors that was responsible for the activation of *miR-23a∼27a∼24-2 cluster* during erythroid differentiation, a Transcription Element Search System-mediated (http://www.cbil.upenn.edu/cgi-bin/tess) sequence analysis was performed and revealed two putative GATA sites scattered within the promoter region of human *miR-23a∼27a∼24-2* loci (Figure 1E; detailed information is shown in Supplementary Figure S1). These sites and additional distal GATA-bound regions were also revealed by ChIP sequencing analysis in mouse (14). ChIP-PCR and quantitative ChIP-PCR (ChIP-q-PCR) were used to validate promoter binding and showed that only the −557 site had GATA-1 occupancy in 48 h hemin-treated K562s (Figure 1F). To determine whether GATA-1 would influence the expression of miR-27a and miR-24, the primary and mature transcripts of miR-27a and miR-24 were evaluated in K562s transfected with siRNAs specific to GATA-1 or constructs overexpressing GATA-1 (Figure 1G). Inhibition of GATA-1 repressed *pri-miR-27a∼24* and mature miRNAs by ∼3-fold and ∼2-fold, respectively (Figure 1H), whereas overexpression of GATA-1 enhanced the levels of primary and mature miRNAs (Figure 1H). Furthermore, consistent with the upregulation of GATA-1 during erythroid differentiation (Figure 1I), the −557 GATA-1 site showed ∼2-fold higher precipitation at 48 h than at 0 h in hemin-treated K562s (Figure 1J). These results introduced the possibility that the higher occupancy of GATA-1 following hemin treatment may contribute to miR-27a and 24 activation. To test this hypothesis, the recruitment of pol II to the −557 site was assessed by ChIP analysis. As expected, the presence of pol II increased ∼3-fold at the GATA-1 site at 48 h compared with 0 h (Figure 1J), which is consistent with the increase of miR-27a and 24 during erythroid differentiation.

The activity of GATA-1 on the *miR-23a∼27a∼24-2* promoter was examined by using a luciferase reporter assay following co-transfection with a GATA-1 overexpressing-vector and either a wild-type pGL-3-promoter construct (WT) or a mutant promoter (MUT) in 293T cells. Increased GATA-1 levels successfully increased reporter activity by ∼2-fold, whereas introduction of a mutant promoter abolished this activity. These results suggest the occurrence of GATA-1-directed positive regulation of the *miR-23a∼27a∼24-2 cluster* (Figure 1K).

### MiR-27a and miR-24 promoted erythroid differentiation in CD34+ HPCs

**Figure 2.**
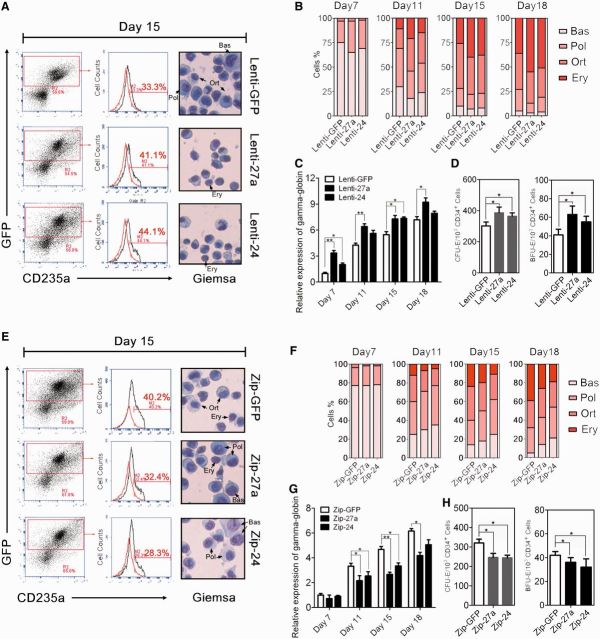
MiR-27a and miR-24 promoted erythroid differentiation in CD34+ HPCs. (**A**) Monitoring of the GFP^+^ population (left panel) and the CD235a stained GFP^+^ fraction (medium panel) of Lenti-miRNA-transduced CD34+ HPCs on day 15 of E culture. The morphology (May-Grunwald Giemsa staining) of CD34+ HPCs derivatives on day 15 is shown in the right panel. A 400X magnification of a representative field is shown. (**B**) Detection of the erythroid differentiation degree of CD34+ HPCs transduced with Lenti-27a, Lenti-24 or Lenti-GFP in E culture at the indicated time. Percentages of basophilic (Bas), polychromatophilic (Pol), orthochromatic (Ort) erythroblasts and erythrocytes (Ery) were determined by May-Grunwald/Giemsa staining of cytospin preparations. (**C**) Q-PCR analysis of gamma-globin mRNA expression in CD34+ HPCs transduced with Lenti-27a, Lenti-24 or Lenti-GFP in E culture at the indicated time. (**D**) A comparison of the erythroid colony-forming capacity (CFU-E, BFU-E) of CD34+ HPCs transduced with Lenti-miRNAs. Error bars represent the standard deviation obtained from three independent experiments. *P < 0.05; **P < 0.01. (**E**) FACS monitoring of CD34+ HPCs transduced with Zip-miRNA or Zip-GFP as described in (A). (**F**) Detection of the erythroid differentiation degree of CD34+ HPCs transduced with Zip-27a, Zip-24 or Zip-GFP as described in (B). (**G**) Detection of gamma-globin mRNA level in CD34+ HPCs transduced with Zip-miRNA. (**H**) Colony-forming assay of CD34+ HPCs transduced with Zip-miRNA as described in (D).

To assess the functional implications of miR-27a and miR-24 in human erythropoiesis, we evaluated the degree of erythroid differentiation in EPO-driven CD34+ HPCs following miRNA transduction (the efficiency of miRNA transduction is shown in Supplementary Figure S2A). The generation of mature erythroid cells (CD235a-positive cells) in GFP-positive subsets was consistently faster for miR-27a- or miR-24-transduced HPCs compared with the Lenti-GFP-transduced control (∼4% for 27a and ∼10% for 24 at Day 11; ∼8% for 27a and ∼11% for 24 at Day 15, Supplementary Figure S2B; Figure 2A). Additionally, these cells demonstrated morphologic evidence of erythroid differentiation following miRNA transduction. As erythroid differentiation proceeded, erythroblasts displayed a gradual decrease in cell size and an increase in chromatin condensation, and these changed were also seen in miR-27a- or miR-24-transduced HPCs compared with the control (Figure 2A). Cell-counting analyses at different stages of differentiation showed an increase of mature erythroblasts (orthochromatic and erythrocyte) in miR-27a- or miR-24-transduced HPCs with a concomitant decrease of immature erythroblasts (basophilic and polychromatic erythroblasts) (Figure 2A and B). The accumulation of gamma-globin level in differentiated erythrocytes also increased on miR-27a or miR-24 transduction compared with the GFP-transduced HPCs (Figure 2C).

Meanwhile, colony formation assays were performed in a methylcellulose medium to assess the erythroid clonogenic capacity of transduced HPCs. The numbers of CFU-E and BFU-E colonies were scored after days 7 and 15. MiR-27a- or miR-24-transduced HPCs produced more than 50% or 30% BFU-Es compared with the GFP-transduced HPCs after 15 days of E culture (Figure 2D). Moreover, the miRNA-transduced HPCs generated larger colonies, when a typical BFU-E generated by GFP-transduced HPCs was ∼30∼60 μm, whereas the miR-27a- or miR-24 colonies were larger than 100 μm (Supplementary Figure S2E).

Conversely, a miRNA loss-of-function study using recombinant lentivirus carrying antisense RNAs specific to miR-27a (Zip-27a) or 24 (Zip-24) (the efficiency of miRNA inhibition is shown in Supplementary Figure S2A) demonstrated impaired erythroid maturation, as revealed by fluorescence-activated cell sorting (FACS) analysis (Supplementary Figure S2B; Figure 2E), morphological analysis (Figure 2E and F), gamma-globin detection (Figure 2G) and colony-forming assays (Figure 2H; Supplementary Figure S2F). Taken together, these results demonstrated that miR-27a and miR-24 were required for the proper erythroid differentiation in primary cultured CD34+ HPCs.

### Suppression of miR-27a or miR-24 blocked erythroid differentiation in zebrafish

**Figure 3.**
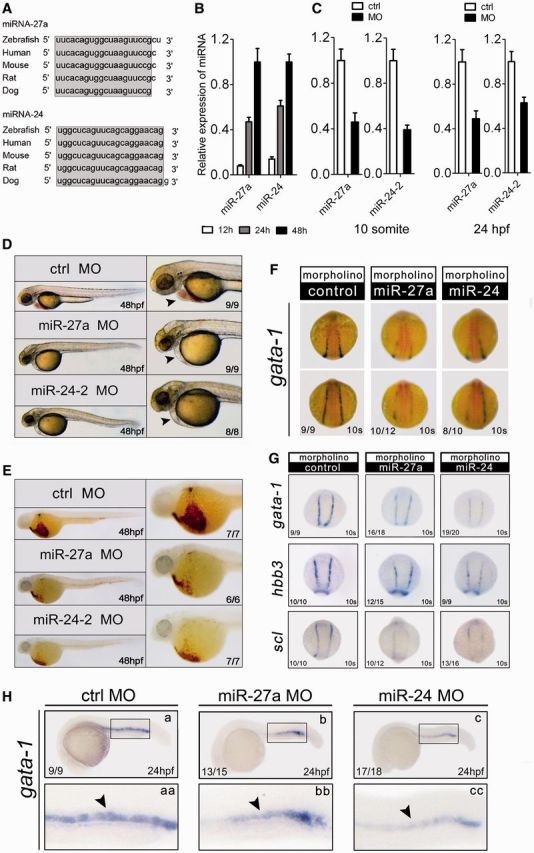
Suppression of miR-27a or miR-24 blocked erythroid differentiation in zebrafish. (**A**) A manual alignment of mature miR-27a and miR-24 genomic sequences in five vertebrate species. Sequences were derived from the miRBase database (Release 18). (**B**) Mature levels of miR-27a and miR-24 at embryo development 12, 24 and 48 h in zebrafish. (**C**) Q-PCR analysis of miR-27a and miR-24 levels, in 10 s and 24 hpf embryos after morpholino injection. (**D**) Lateral view of the heart and yolk sac of a control morpholino (Ctrl MO) or a morpholino antagonist of miRNA (miR-27a MO, miR-24 MO)-injected embryos. (**E**) O-dianisidine staining for haemoglobin in randomly selected 48 h post-fertilization (48 hpf) embryos injected with Ctrl MO, miR-27a MO or miR-24 MO. (**F**) Expression of *gata-1* in 10 somite (10 s) embryos injected with Ctrl MO or miRNA MO by whole mount *in situ* hybridization. Somite boundaries are stained in red. (**G**) Expression of *gata-1, hbbe3* and *scl* in 10 s embryos injected with Ctrl MO or miRNA MO by whole mount *in situ* hybridization. (**H**) Expression of *gata-1* in 24 hpf embryos injected with Ctrl MO or miRNA MO by whole mount *in situ* hybridization.

A conservation analysis of miR-27a and miR-24 sequences indicated that they are highly conserved among multiple species, including zebrafish (Figure 3A). Zebrafish demonstrate increased miR-27a and miR-24 levels during development and is a classic and reliable model to study haematopoietic gene function (Figure 3B). To analyse the roles of miR-27a and miR-24 *in vivo*, we used miRNA MOs to test whether the knock-down of endogenous miRNAs would affect zebrafish erythropoiesis. A Q-PCR analysis was performed to test functionality and showed 2- to 3-fold reduction of mature miRNAs in MO-injected embryos (Figure 3C). From 24 hpf onwards, miRNA MO-injected embryos showed a reduction in the number of blood cells in circulation in the presence of a beating heart. Interestingly, there were negligible or fewer blood cells inside the heart and blood vessels at 48 hpf compared with the control embryos (Figure 3D; Supplementary Figure S3A). A previous study reported the effect of miR-24 on zebrafish cardiac development (15). To distinguish whether the loss of blood cells was derived from cardiac deficiency, we used o-dianisidine staining to detect the number of circulating erythrocytes. In control embryos, haemoglobin-positive erythrocytes demonstrated robust staining with o-dianisidine, whereas in miRNA MO-injected embryos, there was a relative lack of staining detected (Figure 3E; Supplementary Figure S3B). Furthermore, the expression of erythroid marker *gata-1* in miRNA MO-injected embryos notably decreased in comparison with that of control embryos at 10 somites (Figure 3F and G) and 24 hpf (Figure 3H). Additionally, a reduction in *hbbe3* and *scl* staining was also observed in miR-27a and miR-24 MOs-injected embryos at 10 somites (Figure 3G), which suggested an impairment of early erythroid differentiation by miRNA MOs treatment. These results suggested that miR-27a and miR-24 are required for erythroid differentiation during primitive haematopoiesis in zebrafish.

### MiR-27a and miR-24 co-targeted GATA-2 in erythrocytes

**Figure 4.**
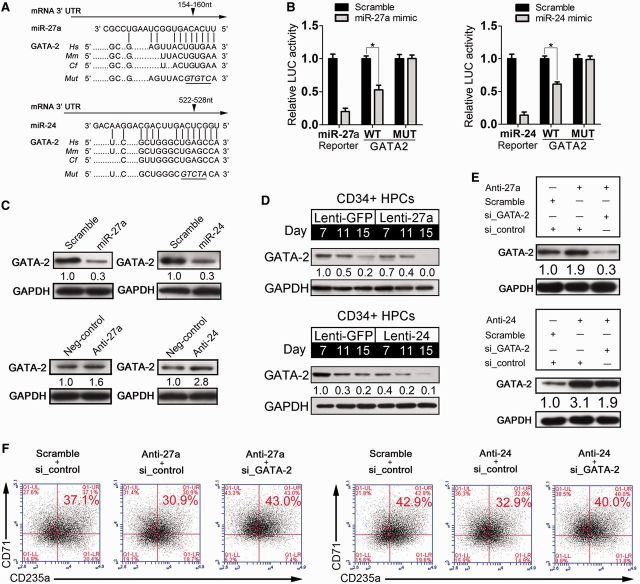
GATA-2 was post-transcriptionally regulated by miR-27a and miR-24 during erythropoiesis. (**A**) A computer prediction of conserved and mutated binding sites within the 3′ UTR of GATA-2 mRNA for miR-27a and miR-24. (**B**) Relative luciferase activity of the indicated GATA-2 reporter constructs. Error bars represent the standard deviation obtained from three independent experiments. *P < 0.05. (**C**) Immunoblot analysis of GATA-2 in K562s transfected with scramble or miRNA mimics (miR-27a, miR-24) or inhibitors (Anti-27a, Anti-24). (**D**) Immunoblot analysis of GATA-2 in CD34+ HPCs transduced with Lenti-GFP control and lentivirus expressing miR-27a or miR-24 (Lenti-27a or Lenti-24). (**E, F**) ‘Rescue’ assays for miRNAs and GATA-2 during erythroid differentiation. Immunoblot analysis of GATA-2 in K562s treated with scramble or Anti-27a/24 (E) for 24 h. These cells were subsequently treated for another 24 h with control siRNAs or siRNAs specific to GATA-2 and were then treated with hemin treatment for 0, 48 and 72 h. (F) FACS analysis of K562s stained for CD71 and CD235a expression after 48 h of hemin induction as described earlier in the text.

In erythropoiesis, GATA-2 acts in opposition to GATA-1. Downregulation of GATA-2 is required for terminal erythroid differentiation (16). Although GATA-1 is known to transcriptionally repress GATA-2 during erythropoiesis (17,18), it is still unknown whether there is a post-transcriptional regulation of the GATA-2 switch-off. A bioinformatic analysis showed that the *GATA-2* 3′ UTR has potential binding sites for both miR-27a and miR-24 (Figure 4A). To confirm the regulatory interaction, we evaluated the effects of miR-27a and miR-24 on reporter genes carrying the *GATA-2* 3′ UTR. As expected, miR-27a and miR-24 reduced luciferase gene activity by ∼50% and ∼30%, respectively. Mutation of these miRNA sites abolished inhibition (Figure 4B). Additionally, the ectopic expression of miR-27a or 24 in K562s reduced GATA-2 levels by ∼3-fold, whereas GATA-2 levels increased by ∼2-fold when endogenous miRNAs were inhibited (Figure 4C). Meanwhile, CD34+ HPCs were transduced with a recombinant lentivirus harbouring miR-27a (Lenti-27a) or 24 (Lenti-24) following days 7, 11 and 15 of E culture. The level of miRNAs in transduced HPCs was measured and demonstrated ∼2- to 4-fold higher levels than the Lenti-GFP control (Supplementary Figure S2A). MiR-27a or miR-24 transduction decreased the protein level of GATA-2 compared with the GFP control (Figure 4D). Thus, our attempt to investigate the regulatory mechanism of miR-27a and miR-24 during erythropoiesis led to the identification of another erythroid GATA member, GATA-2.

### GATA-2 modulated the regulatory effects of miR-27a and miR-24 on erythroid differentiation

To clarify the biological links among miR-27a/24, GATA-2 and erythroid phenotype, we used a ‘rescue’ experiment to assess their functional relevance in differentiating K562s. We first evaluated the role of miR-27a or miR-24 in K562s by using benzidine staining and a gamma-globin level measurement. As expected, the percentage of benzidine-positive cells (Supplementary Figure S4A) and gamma-globin accumulation (Supplementary Figure S4B) increased in miR-27a or miR-24 over-expressed K562s, whereas the percentage of benzidine-positive cells decreased in K562s following the inhibition of miR-27a or miR-24. After performing a rescue assay, a 2- to 3-fold increase in GATA-2 protein levels was observed after anti-miRNA treatment (Figure 4E). This led to a decrease in gamma-globin accumulation (Supplementary Figure S6) and differentiated erythroid population (Figure 4F). Furthermore, the addition of siRNAs specific to GATA-2 following anti-miRNA treatment resulted in the inhibition of GATA-2 protein compared with control siRNAs (Figure 4E). In contrast to decreased GATA-2 protein levels, an increase in gamma-globin accumulation (Supplementary Figure S5) and in the percentage of differentiated erythroid cells (Figure 4F) was observed. These data suggested that the inhibition of GATA-2 could rescue the erythroid deficiency caused by miR-27a or miR-24 silencing.

### The *miR-23a∼27a∼24-2 cluster* was regulated by GATA1/2 switch during erythroid differentiation

**Figure 5.**
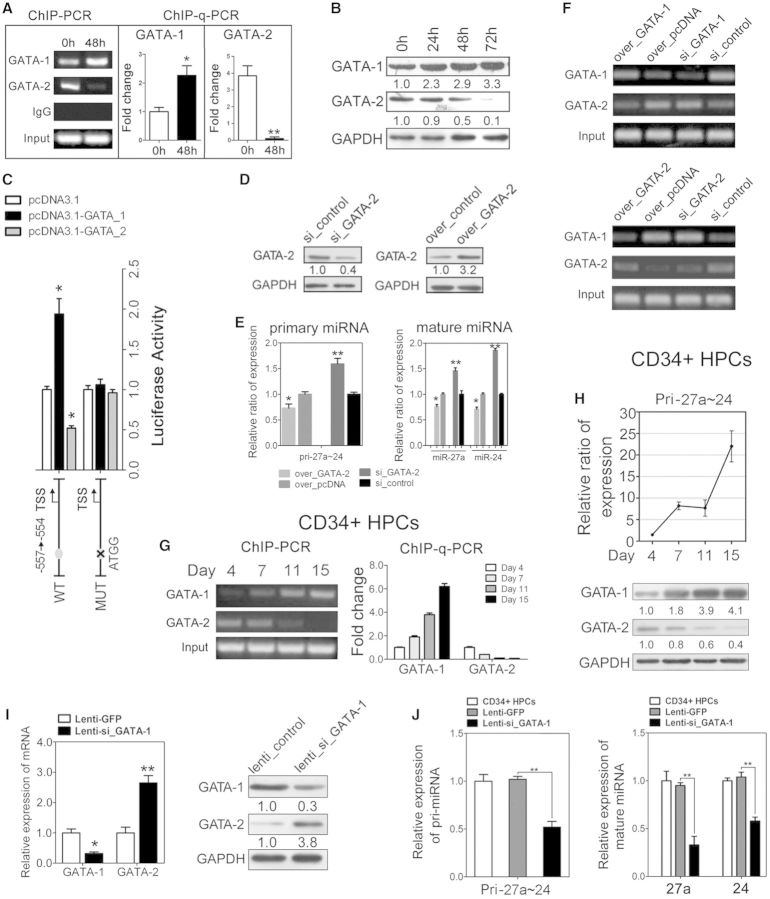
The GATA switch regulated miR-27a and miR-24 expression. (**A**) ChIP-PCR and ChIP-q-PCR analysis of GATA-1, GATA-2 and Pol II occupancy at the −557 site in hemin-treated K562s at 0 and 48 h. ChIP-q-PCR results are shown as fold enrichment compared with input. Error bars represent the standard deviation obtained from three independent experiments. *P < 0.05; **P < 0.01. (**B**) Immunoblot analysis of GATA-1 and GATA-2 expression in K562s undergoing erythroid differentiation for 0, 24, 48 and 72 h. (**C**) Functional activity of GATA-1 and GATA-2 on the wild-type GATA site (WT) or mutant site (MUT) of the *miR-23a∼27a∼24* promoter in a luciferase reporter analysis. Error bars represent the standard deviation obtained from three independent experiments. *P < 0.05. (**D**) Immunoblot analysis of GATA-2 in K562s treated with siRNAs specific to GATA-2 (si_GATA-2) for 48 h or K562s transfected with a construct overexpressing GATA-2 (over_GATA-2) for 48 h, respectively. (**E**) Q-PCR analysis of Pri-27a∼24, mature miR-27a and miR-24 expression in K562s treated as described in (D). (**F**) ChIP-PCR analysis of GATA-1 and GATA-2 occupancy at the −557 site in K562s transfected with control siRNAs or siRNAs specific to GATA-1/GATA-2 or K562s transfected with empty pcDNA3.1 vectors or pcDNA3.1_GATA-1/GATA-2. (**G**) ChIP-PCR and ChIP-q-PCR analysis of GATA-1 and GATA-2 occupancy at the −557 site in CD34+ HPCs of E culture. (**H**) Q-PCR analysis of Pri-27a-24 abundance in CD34+ HPCs of E culture (Top panel). Error bars represent the standard deviation obtained from three independent experiments. An immunoblot analysis of GATA-1 and GATA-2 expression in CD34+ HPCs of E culture (Bottom panel). (**I**) Q-PCR (left panel) and immunoblot (right panel) analysis of GATA-1 and GATA-2 expression in CD34+ HPCs transduced with lentivirus-expressing siRNAs against GATA-1 or control on day 11 of E culture. Error bars represent standard deviation obtained from three independent experiments. *P < 0.05; **P < 0.01. (**J**) Q-PCR analysis of Pri-27a∼24, mature miR-27a and miR-24 expression in CD34+ HPCs treated as described in (I). Error bars represent the standard deviation obtained from three independent experiments. **P < 0.01.

Given the aforementioned observations that GATA-1 could reside on the −557 promoter site of *miR-23a∼27a∼24-2 cluster* and activate transcription, we attempted to determine whether GATA-2 and GATA-1 share the same −557 binding site but yield different biological outputs. A ChIP-q-PCR analysis in differentiated K562s was conducted with antibodies specific to GATA-1 or GATA-2 and revealed a selective increase of GATA-1 (∼3-fold) but a reduction of GATA-2 occupancy (∼10-fold) at this site in differentiated K562s (Figure 5A). This result was consistent with the expression level of GATA-1 and GATA-2 in erythroid-differentiated K562s (Figure 5B). The activity of these two GATA factors at the −557 site was subsequently examined using a luciferase reporter assay following co-transfection with a GATA-1- or GATA-2-overexpressing vector and a pGL-3 miRNA promoter construct carrying either the wild-type GATA site (WT) or a mutant one (MUT) in 293T cells. The introduction of the GATA-1 protein increased reporter activity by ∼2-fold, whereas the GATA-2 protein decreased the activity by ∼2-fold; however, the mutant promoters abolished the effects of both GATA factors (Figure 5C). To further establish the connection between GATA-2 and miR-27a/24, the levels of both the primary and mature *miR-23a∼27a∼24-2 clusters* were evaluated in K562s transfected with either siRNAs specific to GATA-2 or constructs over-expressing GATA-2 (Figure 5D). Increased GATA-2 expression led to a decrease in the levels of Pri-27a∼24 and mature miR-27a or miR-24 (Figure 5E), whereas GATA-2 knock-down increased the transcription and maturation of miR-27a and miR-24 (Figure 5E). These results suggest that GATA-2-dependent *miR-23a∼27a∼24-2 cluster* repression occurs.

Because a change in GATA factor expression is a critical determinant of the switch, we further investigated whether this GATA switch could be affected by the manipulation of GATA factor levels in erythroid cells. As expected, increased GATA-1 expression in K562s led to an increase of GATA-1 occupancy at the −557 site but led to less binding of GATA-2. Additionally, reduced GATA-1 expression resulted in decreased GATA-1 occupancy and enhanced GATA-2 binding (Figure 5F). These binding changes resulted in transcriptional changes of miR-27a and miR-24, as evidenced by an increase or decrease in their primary and mature transcripts on GATA-1 over-expression or silencing (Figure 1H). In contrast, the loss of GATA-2 expression facilitated GATA-1 replacement at the −557 site, whereas ectopic GATA-2 expression reduced this switch (Figure 5F). These results were consistent with the expression levels of miR-27a and miR-24 (Figure 5E).

Consistent with the results observed in K562s, a differentiation stage-specific increase of GATA-1 occupancy with a concomitant reduction of GATA-2 occupancy was revealed in primary HPCs undergoing E culture (Figure 5G). The change of GATA factor occupancy was in parallel with their expression (Figure 5H, bottom panel) during HPCs erythroid differentiation and the accumulation of Pri-miR-27a∼24 (Figure 5H, top panel). The effect of GATA-1 on miR-27a and miR-24 expression in HPC erythroid differentiation was examined. A lentivirus with GATA-1 shRNA was used to infect CD34+ HPCs for 11 days in E culture. Q-PCR and immunoblotting were performed to measure the RNA (Figure 5I, left panel) and protein levels (Figure 5I, right panel), and both showed repression of GATA-1 expression and activation of GATA-2 expression. The levels of pri-miR-27a∼24 and mature miRNAs in these HPCs were then examined. As expected, there was a ∼2- to 3-fold reduction in both the primary transcript (Figure 5J, left panel) and mature level (Figure 5J, right panel) of miR-27a and 24 in CD34+ HPCs transduced with GATA-1 shRNAs compared with the control shRNAs.

Based on the aforementioned observations, we propose that the GATA1/2 switch at the *miR-23a∼27a∼24-2* promoter is responsible for their upregulation during erythropoiesis.

### MiR-27a and miR-24 mediated a forward regulatory circuit composed of a GATA switch

**Figure 6.**
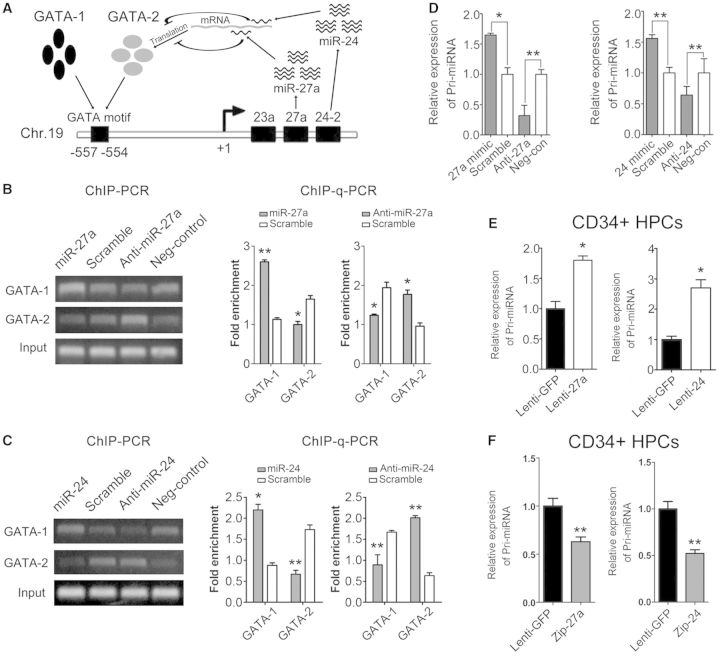
A regulatory circuit involving GATA-1, GATA-2 and miR-27a/24 in erythropoiesis. (**A**) A schematic representation of the regulatory circuit comprised GATA-1, GATA-2, miR-27a and miR-24 in erythroid differentiation. (**B**, **C**) ChIP-PCR and ChIP-q-PCR analysis of GATA-1 and GATA-2 occupancy at the −557 site in K562s transfected with scramble or miRNA mimics and inhibitors (B, r miR-27a and C, miR-24). (**D**) Q-PCR analysis of Pri-27a∼24 abundance in K562s treated as described in (B) and (C). Error bars represent the standard deviation obtained from three independent experiments. *P < 0.05; **P < 0.01. (**E**) Q-PCR analysis of Pri-27a∼24 abundance in CD34+ HPCs transduced with Lenti-miR-27a/24 or Lenti-GFP control for 11 days. Error bars represent the standard deviation obtained from three independent experiments. *P < 0.05. (**F**) Q-PCR analysis of Pri-27a∼24 abundance in CD34+ HPCs transduced with Zip-miRNA or Zip-GFP for 11 days. Error bars represent the standard deviation obtained from three independent experiments. **P < 0.01.

The aforementioned experiments suggested that in addition to effecting erythroid differentiation, upregulated GATA-1 bound and activated the *miR-27a* and *miR-24* genes, which led to further repression of GATA-2 translation and facilitated GATA-1 replacement of GATA-2 at miRNAs promoter (Figure 6A). Thus, a feed-forward circuit containing miR-27a/24, GATA-2 and GATA-1 may positively regulate erythroid differentiation. To confirm this model, we analysed the association of GATA-2 and GATA-1 with the −557 site of the *miR-23a∼27a∼24-2 cluster* following miRNA treatment. Over-expression of miR-27a or miR-24 decreased the binding of GATA-2 and increased GATA-1 occupancy (Figure 6B and C). Similarly, inhibition of miR-27a or miR-24 resulted in increased GATA-2 occupancy and decreased GATA-1 binding with DNA sequences (Figure 6B and C). Therefore, changes in *miR-23a∼27a∼24-2 cluster* transcription would be able to be predicted by disruption of this feedback loop. To test this hypothesis, we evaluated whether breaking the regulatory circuit through over-expression or inhibition of miRNAs could lead to transcriptional changes of this cluster. As expected, treatment with miR-27a or miR-24 mimics increased the level of primary transcript in K562s, whereas repression of miR-27a or miR-24 by miRNA inhibitors decreased the level of pri-miRNA (Figure 6D). Additionally, the level of pri-miRNA was upregulated by ∼2- to 3-fold on day 7 of erythroid cultured HPCs transduced with Lenti-27a or Lenti-24 compared with Lenti-GFP (Figure 6E). Conversely, transduction of Lenti-Zip-27a or Lenti-Zip-24 decreased the primary transcript level of pri-miRNA by ∼2-fold in HPCs on day 7 of E culture (Figure 6F). Overall, the aberrant miR-27a or miR-24 levels fed back to positively modulate the level of their own primary transcripts.

### Enforced expression of miR-27a and miR-24 in mouse enhanced mature erythroid populations

**Figure 7.**
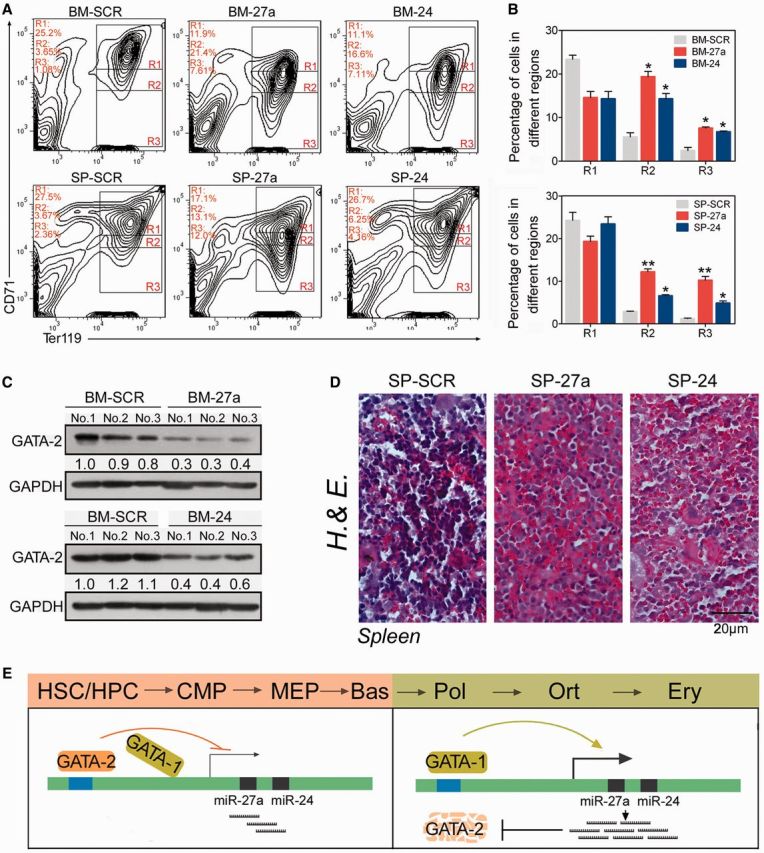
MiR-27a and miR-24 overexpression enhanced erythropoiesis in mice. (**A**) FACS analysis of bone marrow (BM) and spleen (SP) harvested from mice at 8 weeks post-transplantation stained for CD71 and TER119. Representative FACS plots from one mouse were shown. (**B**) Quantitation of the percentage of cells gated within each region in (A). Data from control (SCR, n = 3), miR-27a (n = 3) and miR-24 (n = 3) animals are shown as the means ± SD. *P < 0.05; **P < 0.01. (**C**) Immunoblot analysis of GATA-2 on bone marrow protein harvested from mice at 8 weeks post-transplantation. Three individual animals from each group were run in adjacent lanes. (**D**) Hematoxylin and eosin-stained sections of spleens harvested from mice at 8 weeks post-transplantation. A 400X magnification of a representative field is shown. (**E**) A schematic representation of the regulatory circuit comprised GATA-1, GATA-2, miR-27a and miR-24 in erythroid differentiation.

To test the roles of miR-27a and miR-24 *in vivo*, we conducted transplantation experiments in mice. Scramble or miRNA-transduced bone marrow cells were transplanted into lethally irradiated NOD/SCID mice. Engraftment into recipient mice was confirmed through the analysis of miRNA expression levels of bone marrow and spleen after 8 weeks (Supplementary Figure S6A and B). To determine the effect of miR-27a and miR-24 on erythrocyte differentiation in adult haematopoietic tissues, a flow cytometry analysis was performed 8 weeks post-transplantation. Animals that displayed miR-27a and miR-24 overexpression demonstrated an increase in region 3 (R3) of CD71^low^/TER119^high^ erythrocytes and a concomitant decrease in region 1 (R1) of CD71^high^/TER119^high^ erythroblasts from bone marrow and spleen (Figure 7A, B). This change likely contributed to the observed increase in haematocrit in miRNA-transduced animals compared with the controls (Supplementary Figure S6C).

It is likely that the increased erythroid differentiation observed in miRNA-transduced animals could result from reduced GATA-2 expression. To verify this hypothesis, an immunoblot analysis was performed to determine the GATA-2 level in bone marrow cells of mice that were transplanted with miRNA-expressing cells. Of note, transduction of miR-27a or miR-24 resulted in a ∼2- to 4-fold decrease in the GATA-2 protein level compared with controls (Figure 7C). A histological analysis of spleens revealed a decrease in the erythroid cell clusters in miRNA-transduced animals, which indicated an increase in terminal erythroid maturation (Figure 7D). Therefore, miR-27a and miR-24 accelerated the development of mature erythroid populations by repressing GATA-2 expression in transplanted mouse models.

## DISCUSSION

Recent discoveries have revealed that GATA families are essential regulators in the control of haematopoiesis. GATA-1, GATA-2 and GATA-3 are termed the haematopoietic GATA factors due to their important activities to control distinct and overlapping aspects of haematopoiesis (12,19–21). Interestingly, GATA-1 and GATA-2 often act in opposition on shared sites and co-regulate target genes through a GATA switch during erythropoiesis (22). This occupancy switch from GATA-2 to GATA-1 is in parallel with their reciprocal expression patterns in erythroid differentiation. The GATA switch commonly occurs at numerous loci with critical functions in erythropoiesis such as *α-globin, β-globin* and *alas2* loci, as well as the *Gata-1* and *Gata-2* loci (23–26). In mouse erythroid cells, GATA-1 was demonstrated to positively regulate a previously identified mmu-miR-451 (5). Meanwhile, Dore *et al.* (6) found that dre-miR-451 could modulate zebrafish erythrocyte maturation via targeting *gata-2*. Their observations suggested the existing of a GATA-1/miR-451/GATA-2 axis in mouse and zebrafish erythropoiesis, although needs further validation. Our results not only identified miR-27a and 24 as novel enhancers of erythropoiesis but also provided a dynamic change of GATA-1/-2 occupancy at *miR-27a∼24-2* gene promoter, which conferred the activation of this locus during erythroid maturation. By the light of nature, we further demonstrated the GATA-1/miR-27a/24/GATA-2 regulatory circuit in human erythroid cells, representing the decoding of an expansive regulatory layer of GATA-1 and GATA-2. In details, GATA-2 localized to chromatin sites of the miRNA promoter and transcriptionally repressed *miR-27a* and *miR-24* in early stage erythroblasts. As erythropoiesis proceeds, GATA-1 level increased, and GATA-1 displaced GATA-2 from their shared binding site, thus leading to transcriptional activation of *miR-27a* and *miR-24* (Figure 6E). This is the first evaluated example that the GATA switch operates on non-coding RNA genes to establish and orchestrate genetic networks driving the erythroid development process.

MiR-27a and miR-24 display completely evolutionary conservation among eukaryotes and are organized in a cluster on chromosome 19 of the human genome. This cluster is composed of three members, miR-23a, miR-27a and miR-24, and has been linked to osteoblast differentiation, angiogenesis, cardiac remodelling, skeletal muscle atrophy and tumorigenesis (27–29). Remarkably, only a few reports have raised concerns about the expression of miR-27a and miR-24 in haematopoiesis (30,31). However, not all findings were in agreement due to different cell models or culturing systems used. Our findings were in consistent with studies using the hemin-treated K562s or EPO-induced CD34+ HPCs to differentiate into mature erythrocytes, revealing the upregulation of miR-23a, miR-27a or miR-24 during erythropoiesis, whereas an activin A-mediated erythroid models reported the inhibitory role of miR-24 in haemaglobin accumulation. With the exception of observations from the activin-induced haematopoietic differentiation model (32), miR-27a and miR-24 have been constantly demonstrated increased accumulation as differentiation proceeds, which support the idea that activation of the *miR-27a* and *miR-24* loci might be required for the terminally differentiated cells. In contrast to miR-451 locus whose expression was restricted to the fetal liver in embryonic day (E) 16.5 mouse embryos, the major site of haematopoiesis and erythropoiesis at this stage of development, miR-27a and miR-24 seem to serve as universal regulators in different cell types. We speculate that miR-27a and miR-24 may serve at a ‘standby state’, which means they are ready for the manipulation by different cellular factors, as GATA-1 in erythropoiesis, c-MYC in tumour metastasis (33), Runx2 in osteoblast differentiation (28) and PU.1 in B-cell development (34). Thus, the miR-23a∼27a∼24-2 cluster could coordinate with different TFs to determine or maintain such cell fates.

In this study, we demonstrated the co-regulation of *miR-27a∼24* and *Gata-2* transcription by GATA-1 and the co-regulation of GATA-2 production by GATA-1 and miR-27a/24. On a large scale, the transcriptional co-regulation of miRNA and its targets may be prevalent in human genomes, as shown in a computational analysis using gene expression data (35). Recent studies have indicated that the action of miRNA and transcription factors is often coordinated (36), which confirms the coordinated features of the transcriptional and post-transcriptional layers of regulation. Another recent study also indicated the suppression of *GATA-2* by miR-27a on earlier stages of blood differentiation, which forcefully supports our findings in haematopoiesis (37). Here, miR-27a and miR-24 perform post-transcriptional protection through repressing the translation of *GATA-2*, which should not be expressed in differentiated erythroid cells. Furthermore, with respect to on/off regulation by transcription factors, the introduction of a miRNA-mediated repression pathway may provide more elaborate modulation by keeping the protein levels in a specific range.

During development, gene expression is tightly controlled by dynamic regulatory circuits that determine and maintain specific cell lineages. Among these regulatory circuits, miRNA-mediated circuits play an essential role in ensuring properly spatial and temporal gene expression. The regulatory pattern by which transcription factors regulate miRNA and a set of target genes has been revealed to be a recurrent motif that is used to enhance gene regulation in mammalian genomes. Our work identified a critical regulatory loop that includes GATA-1, miR-27a, miR-24 and GATA-2 and characterized their roles and relationship in this feed-forward circuit that underlies erythropoiesis.

## SUPPLEMENTARY DATA

Supplementary Data are available at NAR Online.

## FUNDING

National Key Basic Research Program of China [2011CBA01100 to J.Y.]; National Natural Science Foundation of China [31040021 to J.Y., 31200977 to F.W.]; IBMS, CAMS [2009RC03 to J.Y., 2010PYB06 to J.Y.]; Beijing Municipal Science & Technology Commission [2010B071 to J.Y.]. Funding for open access charge: the National Key Basic Research Program of China [2011CBA01100 to J.Y.].

*Conflict of interest statement*. None declared.

## Supplementary Material

Supplementary Data
