# Editor's Note on ‘A regulatory circuit comprising GATA1/2 switch and microRNA-27a/24 promotes erythropoiesis’

**DOI:** 10.1093/nar/gkaf208

**Published:** 2025-03-13

This is an Editor's Note regarding: Fang Wang, Yong Zhu, Lihua Guo, Lei Dong, Huiwen Liu, Haixin Yin, Zhongzu Zhang, Yuxia Li, Changzheng Liu, Yanni Ma, Wei Song, Aibin He, Qiang Wang, Linfang Wang, Junwu Zhang, Jianxiong Li, Jia Yu, A regulatory circuit comprising GATA1/2 switch and microRNA-27a/24 promotes erythropoiesis, Nucleic Acids Research, Volume 42, Issue 1, 1 January 2014, Pages 442–457, https://doi.org/10.1093/nar/gkt848

In December 2024, the Editors were informed of three concerns regarding image duplication in Figures 1J and 5A, Figures 1I and 5B, and Figures 5F and 6C as outlined below.

**Figure 1.**
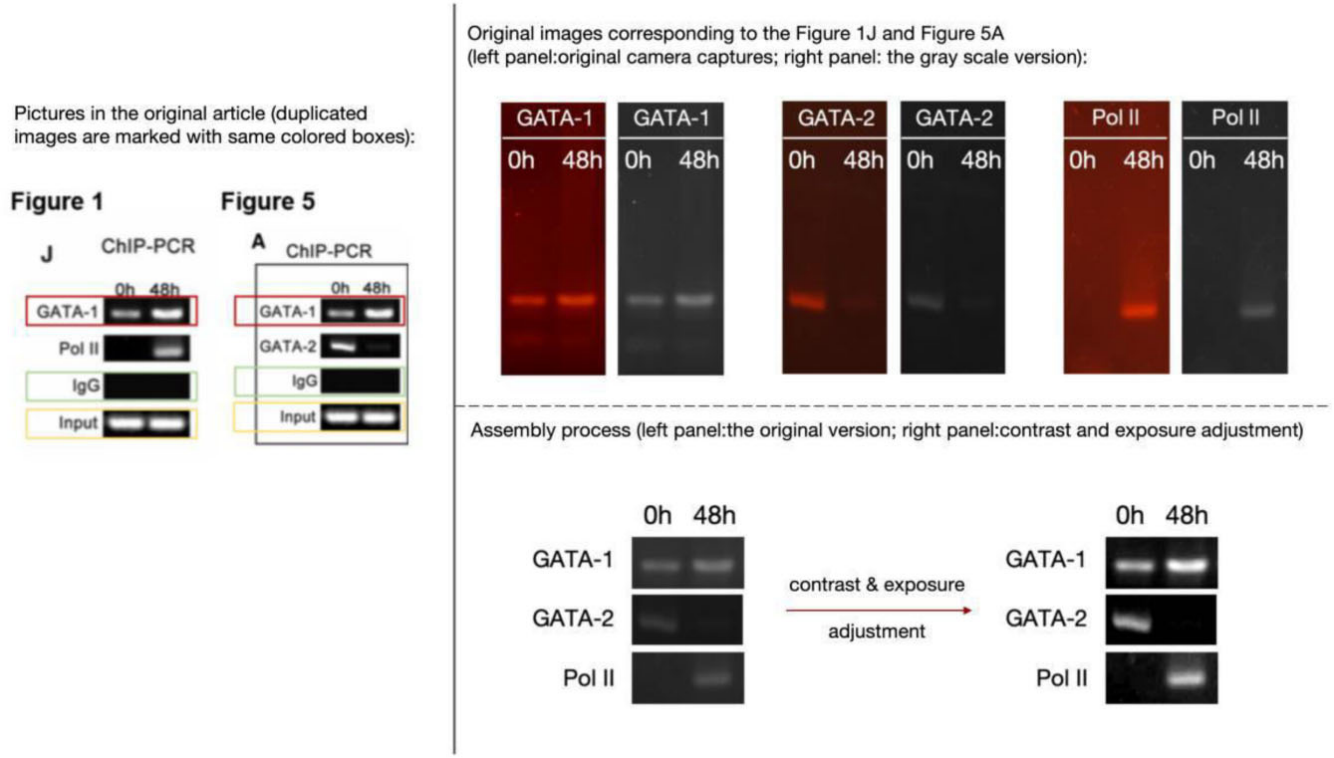
The original GATA-1 images used in Figures 1J and 5A.

**Concern 1**. The GATA-1, IgG and Input strips in Fig.1J appear to have been reused in Fig. 5A.

The authors have supplied the original gel electrophoresis images for GATA-1 (Fig. 1). The authors could not locate the original data for IgG and Input but have supplied images from a separate replicate experiment, which includes results for GATA-1, Pol II, IgG, and Input and corroborates the findings (Fig. 2).

**Figure 2.**
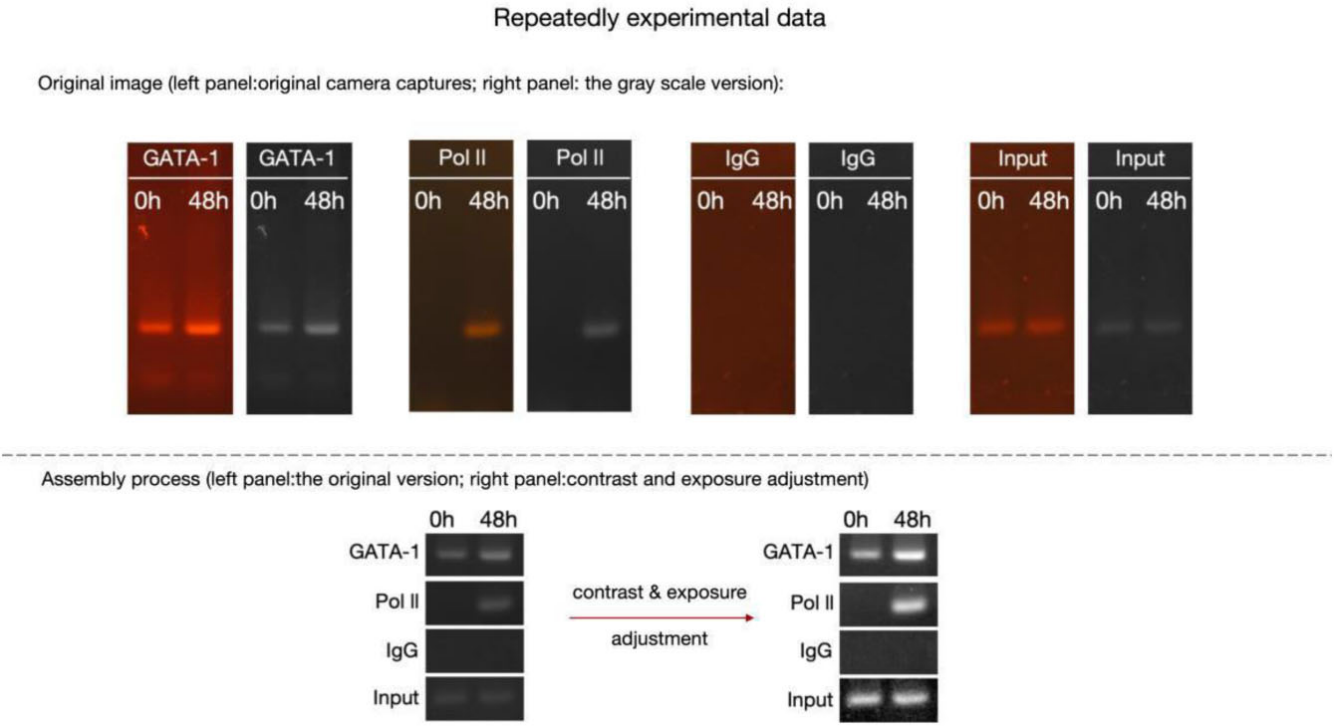
Repeat experimental data for IgG and Input in Figures 1J and 5A.

The authors have also explained the identical images for GATA-1 and IgG in Figures 1J and 5A come from the same ChIP-PCR experiment. To organize the article more logically and to ensure experimental results from the same samples were displayed together, they placed the ChIP-PCR results for GATA-1 and Pol II in Fig. 1, and the ChIP-PCR results for GATA-2 in Fig. 5. To further illustrate and directly compare the dynamic changes in GATA-1, GATA-2, and Pol II binding upstream of the miR-27a/24 locus, they included the results for GATA-1 and Pol II again in Fig. 5. While the Editors confirm that this type of panel duplication is acceptable, it should have been made explicit in the figure legends.

**Concern 2**. The GATA-1 and GAPDH strips in Fig. 1I appear to have been reused Fig. 5B.

The authors have supplied the original gel electrophoresis images for GATA-1 (Fig. 3). The authors could not locate the original data for GAPDH but have supplied images from a separate replicate experiment, which includes results for GATA-2 and GAPDH and corroborates the findings (Fig. 4).

**Figure 3.**
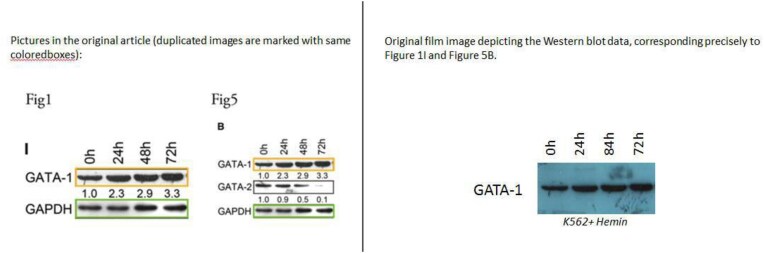
The original GATA-1 images used in Figures 1I and 5B.

**Figure 4.**
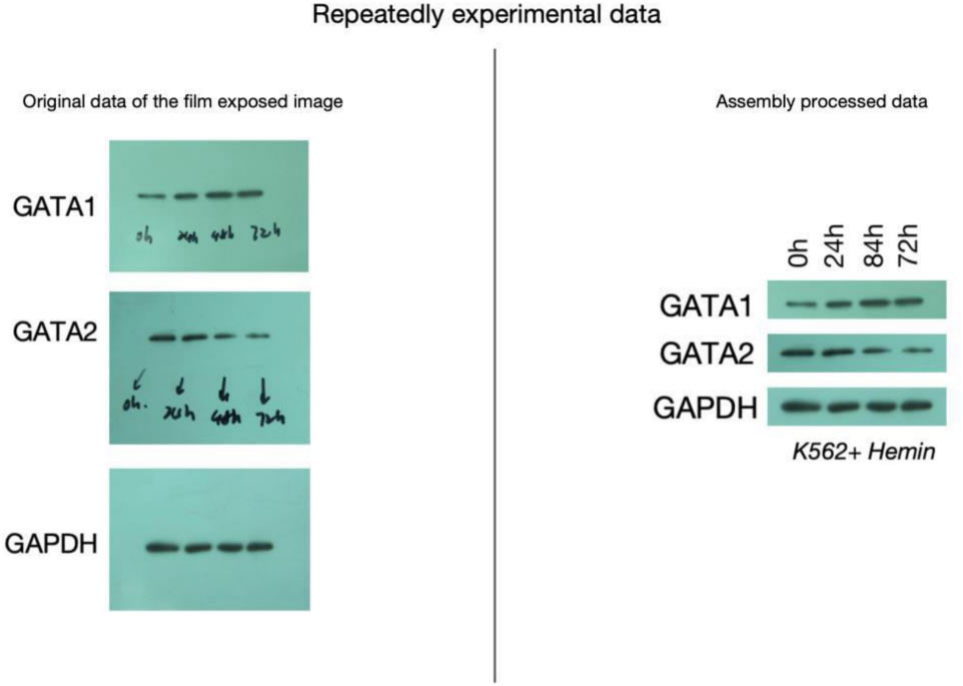
Repeat experimental data for GAPDH in Figures 1I and 5B.

The authors have explained the repeated presentation of GATA-1 and GAPDH results in Figures 1I and 5B is due to a similar rationale as described above. Both sets of results originate from the same experiments using the same samples. This repetition was intended to provide a logical and clear illustration of the dynamic changes observed during the study. While the Editors confirm that this type of panel duplication is acceptable, it should have been made explicit in the figure legends.

**Figure 5.**
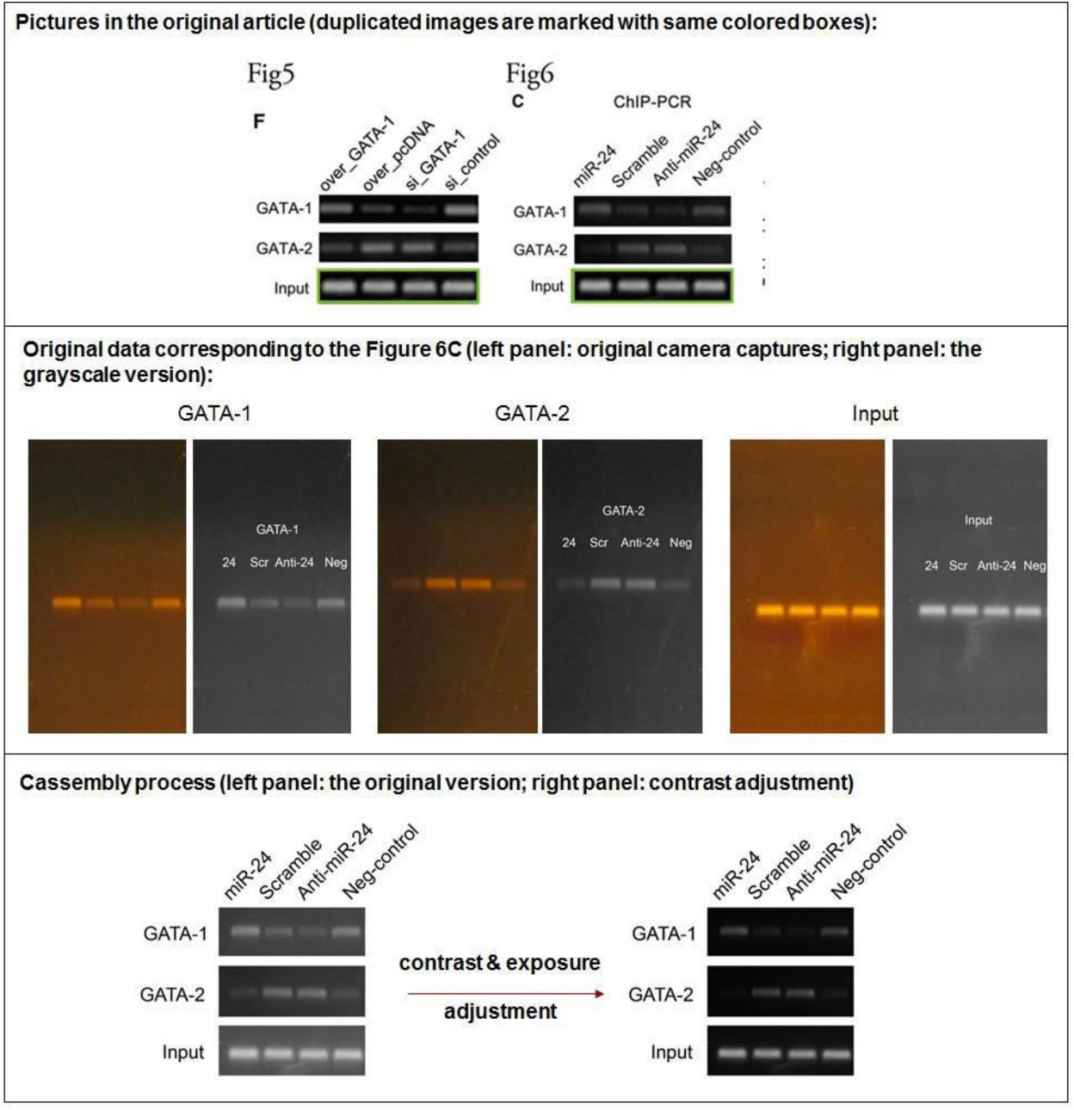
The original GATA-1, GATA-2 and Input images used in Fig. 6C.

**Concern 3**. The Input strip in Fig. 5F appear to have been reused Fig. 6C.

**Figure 6.**
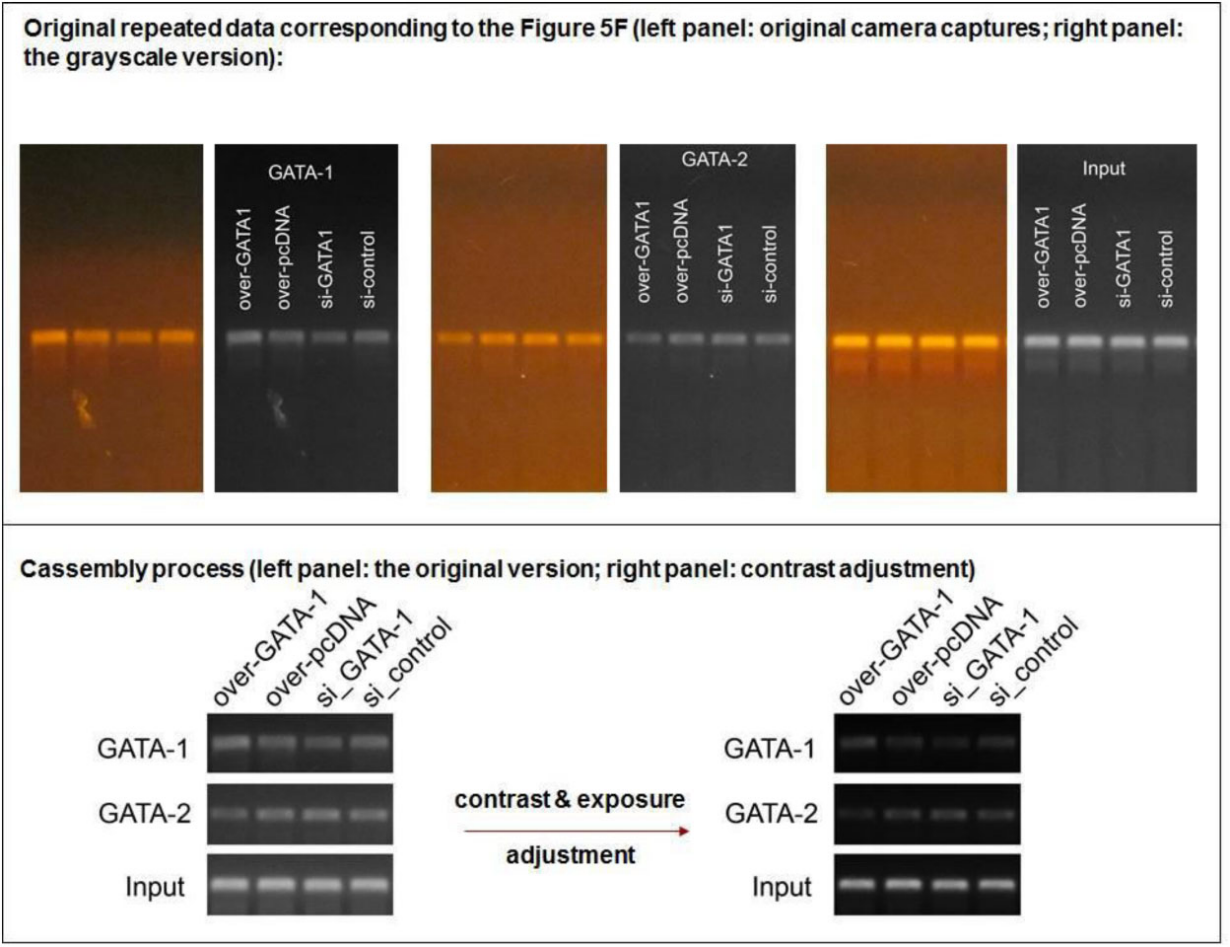
Repeat experimental data for Input in Fig. 5F.

The authors have supplied the original gel electrophoresis images for GATA-1, GATA-2 and Input used for Fig. 6C (Fig. 5). The authors could not locate the original data for Fig. 5F but have supplied images from a separate replicate experiment which corroborates the findings (Fig. 6).

The authors acknowledge that the Input panels presented in Figures 5F and 6C were obtained from two separate experiments, and different Input controls should have been used. The authors believe this accidental duplication occurred during image acquisition, processing, or file management.

In conclusion, while these issues may not impact the results or conclusion of the study, in the absence of some original data, the Editors advise readers to examine Fig. 5 with care.

**Julian E. Sale and Barry L. Stoddard**

Senior Executive Editors

