# Supplementary material for: A regulatory circuit comprising GATA1/2 switch and microRNA-27a/24 promotes erythropoiesis
Source: Nucleic Acids Res. 2013 Sep 18;42(1):442–57. doi: 10.1093/nar/gkt848 (PMC3874166; doi:10.1093/nar/gkt848)
Supplement: Supplementary Data [file supp_42_1_442__index.html]

A regulatory circuit comprising GATA1/2 switch and microRNA-27a/24 promotes erythropoiesis — A regulatory circuit comprising GATA1/2 switch and microRNA-27a/24 promotes erythropoiesis — Supplementary Data 

# A regulatory circuit comprising GATA1/2 switch and microRNA-27a/24 promotes erythropoiesis

## Supplementary Data

files

**Files in this Data Supplement:**

- Supplementary Data - doc file
- Supplementary Data - xls file
