# Supplementary material for: A regulatory circuit comprising GATA1/2 switch and microRNA-27a/24 promotes erythropoiesis
Source: Nucleic Acids Res. 2013 Sep 18;42(1):442–57. doi: 10.1093/nar/gkt848 (PMC3874166; doi:10.1093/nar/gkt848)
Supplement: Supplementary Data [file supp_gkt848_nar-01187-x-2013-File009.doc]

**Supplemental Figures**


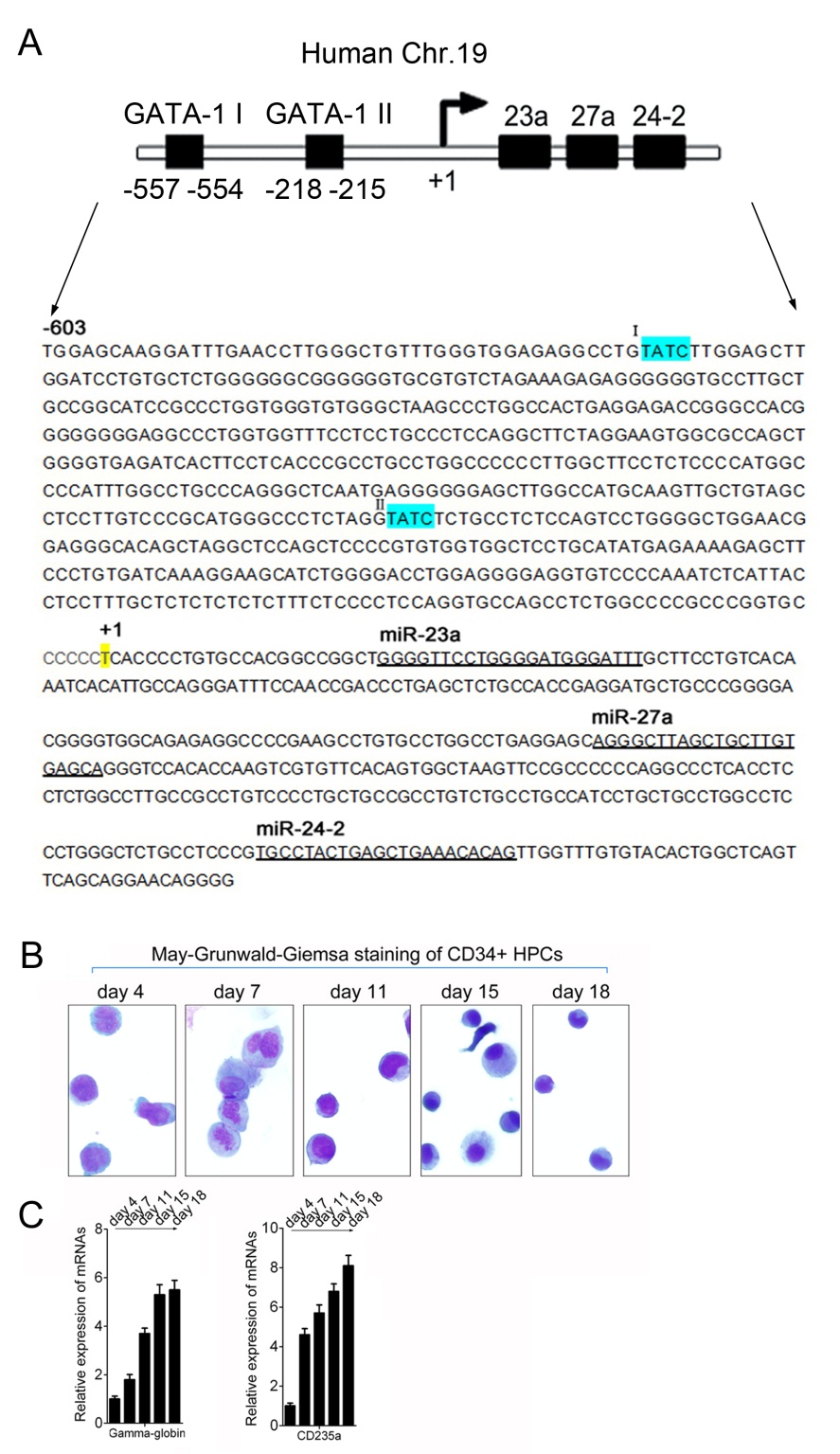


**Figure S1 GATA-1 resides the promoter of *miR-23a~27a~24-2 cluster***

(A) Sequence analysis of GATA motif in the promoter region of miR-23a~27a~24-2 cluster by TESS program. (B) The morphology (May-Grunwald Giemsa staining) of CD34+ HPCs in E culture at the indicated time. A 400X magnification of a representative field is shown. (C) Q-PCR analysis of gamma-globin and CD235a mRNA expression in CD34+ HPCs in E culture at the indicated time.

**
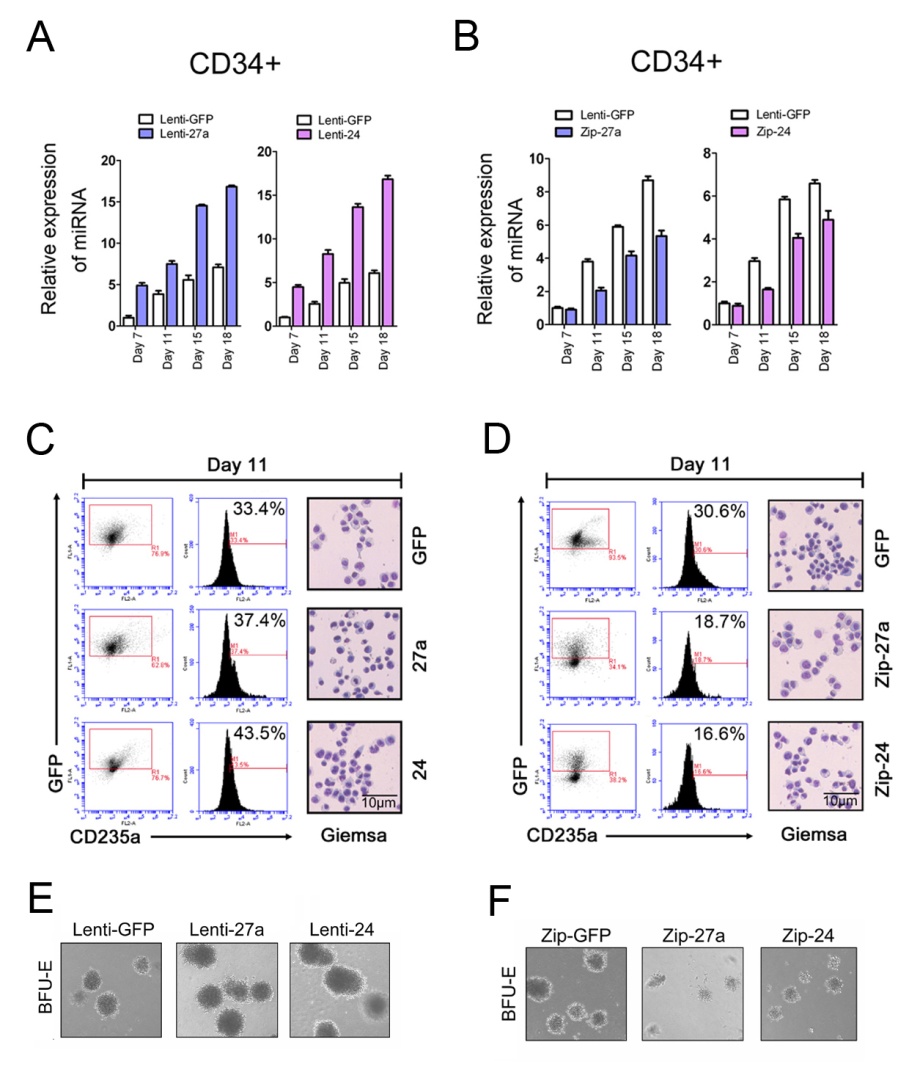
**

**Figure S2 Detection of erythroid differentiation in CD34+ HPCs transduced with lentivirus at day 11 of E culture.**

(A, B) Q-PCR analysis of miR-27a and 24 expression in CD34+ HPCs transduced with Lenti-GFP control, lentivirus expressing miRNAs (Lenti-27a, Lenti-24), or lentivirus expressing antisense oligonucletides to miRNAs (Zip-27a, Zip-24). (C, D) Monitoring of GFP+ population (left panel), and CD235a stained GFP+ fraction (medium panel) of Lenti-miRNA or Zip-miRNA tranduced CD34+ HPCs on day 11 of E culture. Morphology (May-Grunwald Giemsa staining) of CD34+ HPCs derivates on day 11 was shown in the right panel. A 400X magnification of a representative field was shown. (E, F) Representative erythroid-colony forming assays at day 15 of erythroid induction in CD34+ HPCs transduced with Lenti-GFP, Lenti-miRNAs or Zip-miRNAs in semisolid media.


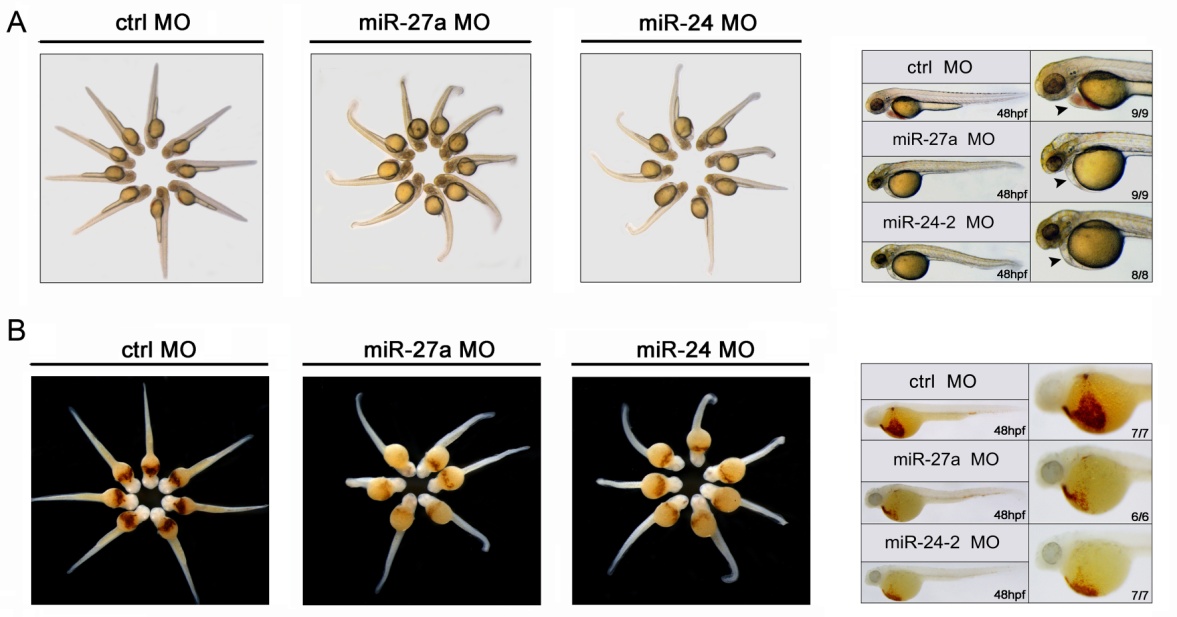


**Figure S3 Functional analysis of miR-27a and 24 in zebrafish**

(A) Lateral view of the heart and yolk sac of control morpholino (Ctrl MO) or morpholino antagonist of miRNA (miR-27a MO, miR-24 MO)-injected embryos corresponding to Figure 3 C. (B) O-dianisidine staining for hemoglobin in randomly-selected 48 hour post fertilization (48 hpf) embryos injected with Ctrl MO, miR-27a MO or miR-24 MO corresponding to Figure 3 D.

**
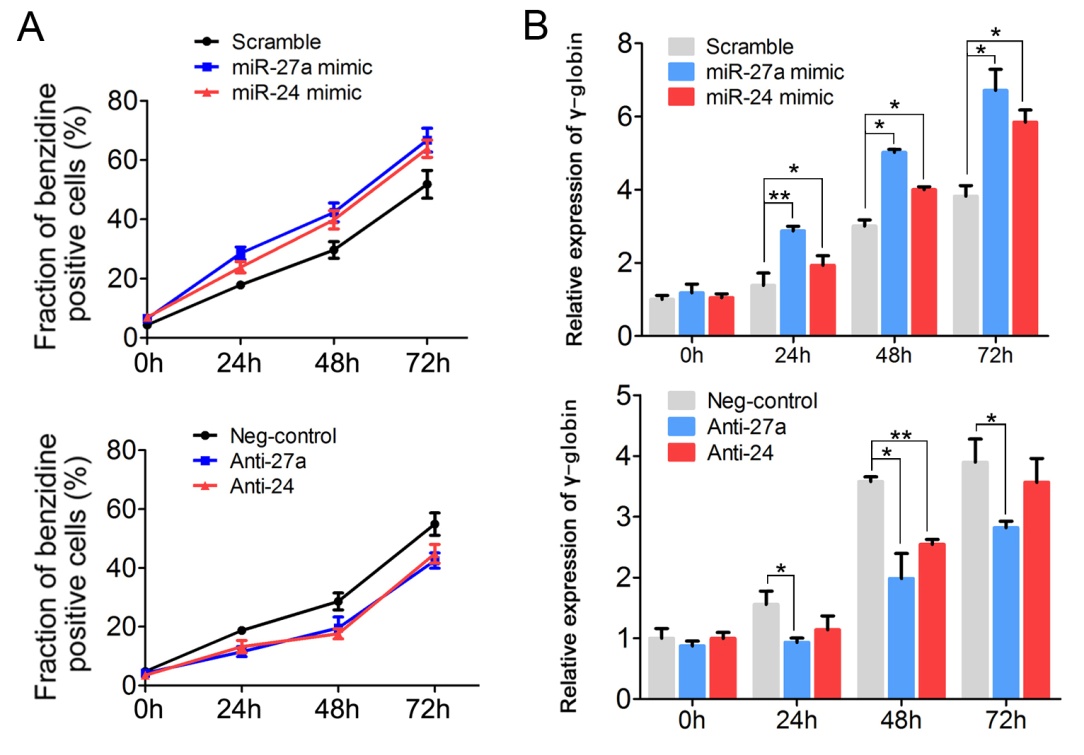
**

**Figure S4 Functional analysis of miR-27a and 24 in K562s**

(A) Benzidine staining of K562s transfected with either miRNA mimics and scramble or Anti-miRNAs and Neg-control for 24h, then following hemin treatment for 0, 24, 48 and 72h. (B) Q-PCR analysis of gamma-globin mRNA expression in K562s as described in (A).


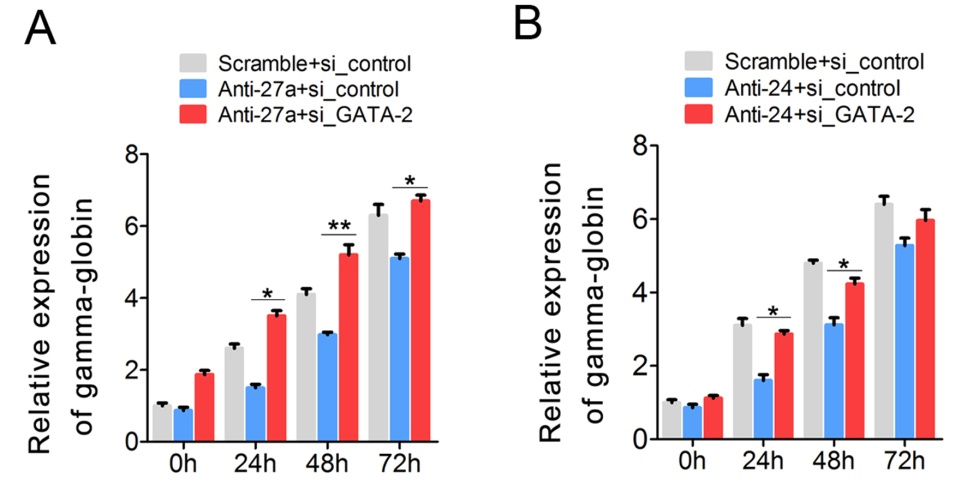


**Figure S5 GATA-2 was involved in miR-27a/ 24-regulated erythropoiesis.**

(A-B) "Rescue" assays for miRNAs and GATA-2 in erythroid differentiation. K562s were treated with scramble or Anti-27a (A), Anti-24 (B) for 24h. These cells were subsequently treated for another 24h with control siRNAs or siRNAs specific to GATA-2, then following hemin induction for 0, 48 and 72h. Q-PCR analysis of gamma-globin mRNA levels in K562s as described above.


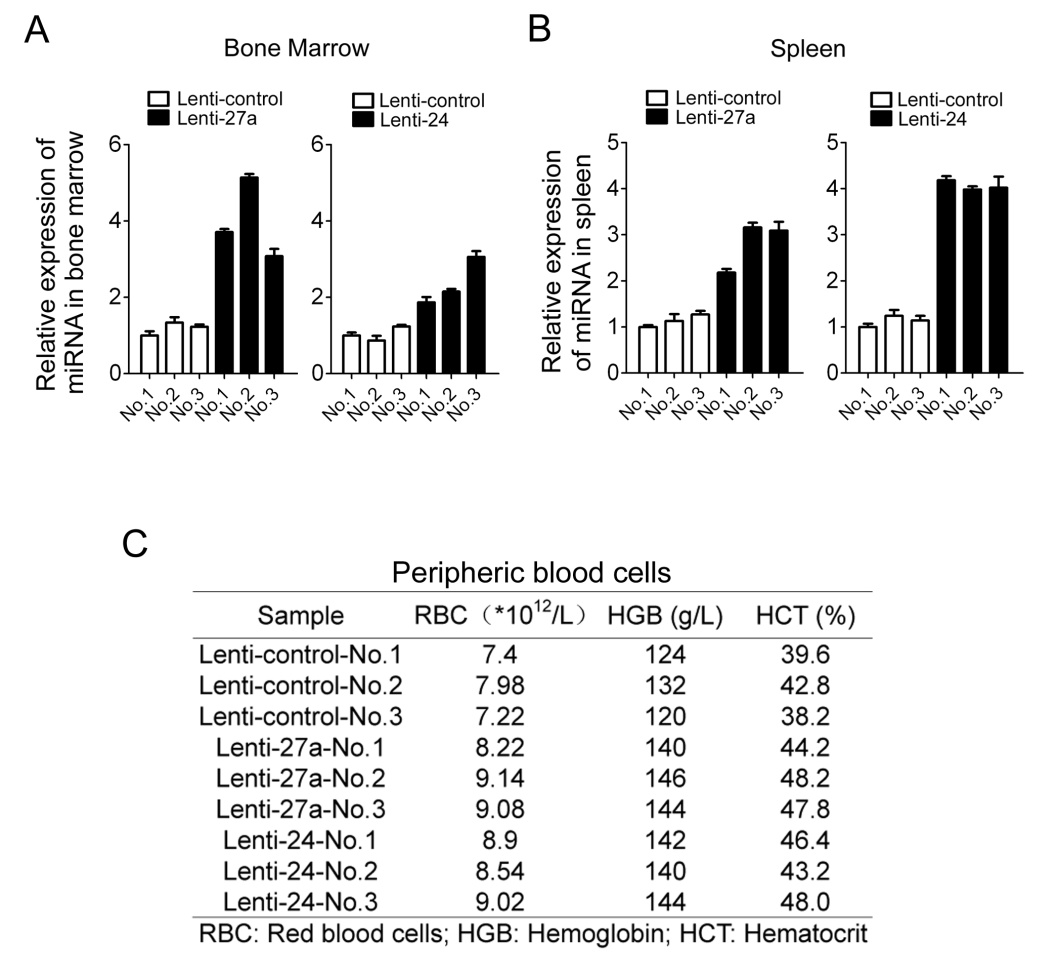


**Figure S6 Evaluation of miR-27a and 24 overexpression on erythropoiesis in mice.**

(A-B) Q-PCR analysis of miR-27a and 24 in bone marrow (A) and spleen (B) from mice at 8 weeks post-transplantation with control or miRNA-transduced bone marrow cells. (C) Complete Blood Count of peripheral [blood](app:ds:Blood) from mice at 8 weeks post-transplantation.
